# Secondary Syphilis Presenting as Glossodynia,* Plaques en Prairie Fauchée*, and a Split Papule at the Oral Commissure: Case Report and Review

**DOI:** 10.1155/2017/1980798

**Published:** 2017-07-12

**Authors:** Walter de Araujo Eyer-Silva, Maria Alessandra Leite Freire, Cecília Angelina Horta-Araujo, Guilherme Almeida Rosa da Silva, Jorge Francisco da Cunha Pinto, Fernando Raphael de Almeida Ferry

**Affiliations:** ^1^Hospital Universitário Gaffrée e Guinle, Centro de Ciências Biológicas e da Saúde, Universidade Federal do Estado do Rio de Janeiro, Rio de Janeiro, RJ, Brazil; ^2^Programa Municipal de HIV/AIDS de Miracema, Posto de Saúde Dr. Irineu Sodré, Av. Nilo Peçanha 59, 28460-000 Miracema, RJ, Brazil

## Abstract

Syphilis has been coined “the great imitator” due to its extreme heterogeneity of presentation and mimicry of other conditions. Therefore, it is essential that physicians be familiar with the full spectrum of its manifestations. Syphilis may also lead to oral lesions that, occasionally, are unaccompanied by concomitant tegumentary findings. Such patients will pose unique diagnostic challenges. We report the case of a 45-year-old HIV-infected male patient in whom secondary syphilis presented with burning mouth and dysgeusia that progressed to glossodynia and odynophagia. Examination revealed painful, shallow erosions on the posterior aspect of the tongue, in a pattern of* plaques en prairie fauchée*. A painful split papule (*fausse perlèche *or false angular cheilitis) was also present in the left commissure. There were no cutaneous lesions. The oral lesions were considered highly suggestive of secondary syphilis. A novel VDRL assay (which was previously negative) yielded a titer of 1/128. Complete clinical remission was rapidly achieved after initiation of penicillin therapy. A comprehensive review of the literature on oral manifestations of syphilis is offered.

## 1. Introduction

Acquired syphilis is a sexually transmitted infection caused by the spirochete* Treponema pallidum*, subspecies* pallidum*. It is a major public health problem worldwide. The disease has been coined “the great imitator” due to its great variability of presentation and mimicry of other conditions. Physicians unaware of its protean manifestations may easily overlook atypical presentations. Moreover, syphilis also leads to oral manifestations and such lesions may be even less likely to suggest the diagnosis [1, 2].

Oral lesions may occur at any of the three main stages of syphilis and the spectrum of manifestations may be mistaken for many other more prevalent disorders. Despite its clinical heterogeneity, oral manifestations can usually be correctly attributed to secondary syphilis when a concomitant skin eruption is present [3, 4]. However, a patient with undiagnosed syphilis may occasionally have only oral lesions [5–14]. These patients will likely pose unique diagnostic challenges. Furthermore, detailed descriptions of oral syphilitic lesions in HIV-infected individuals are scarce [9, 15–19]. We wish to report the case of a 45-year-old HIV-infected male patient in whom the sole manifestations of secondary syphilis were dysgeusia and glossodynia associated with lesions on the dorsal aspect of the tongue and left oral commissure.

## 2. Case Report

A 45-year-old HIV-infected male patient presented with a 4-week history of burning mouth and dysgeusia that progressed to glossodynia over the course of a few days. A 7-day treatment with fluconazole, followed by a 7-day course of itraconazole, had been offered without clinical improvement. The condition worsened to the point of weight loss and feeding difficulties due to sore throat and odynophagia.

The patient was on successful highly active antiretroviral therapy with lamivudine, tenofovir, and efavirenz for more than 4 years. He reported no prior rash and denied the use of drugs other than his current antiretroviral regimen. There was no evidence of a psychiatric disorder and no major complications of AIDS had ever been recorded. He was a current smoker in otherwise good health. Secondary syphilis had been fully and successfully treated 8 years earlier and previous Venereal Disease Research Laboratory (VDRL) assays were negative.

**Figure 1:**
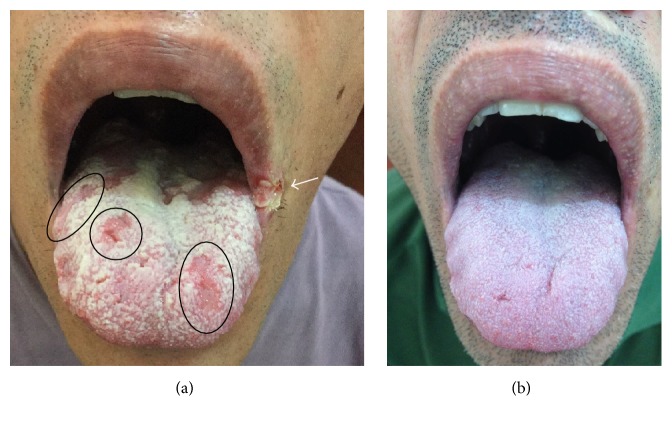
Clinical images of the 45-year-old male patient. (a) Shallow, round to oval depapillary erosions on a background of a whitish, nonwipeable hyperkeratotic thickening of the posterior aspect of the tongue, in a pattern of* plaques en prairie fauchée*. A fibrin-covered commissural papule (split papule) in the left oral commissure is indicated by the arrow. All lesions were very painful. (b) Complete remission after penicillin treatment.

Examination of the oral cavity (Figure 1(a)) revealed shallow, painful, round to oval depapillary erosions on a background of a whitish, nonwipeable hyperkeratotic thickening of the posterior aspect of the tongue. This overall aspect shared resemblance with reported cases of syphilitic lesions of the tongue [11, 18–20], as well as a sign previously described as* plaques en prairie fauchée* [21]. A painful split papule was also present in the left oral commissure. This commissural lesion was not a simple fissure as seen in angular cheilitis* (perlèche)*. Instead, it was a fibrin-covered commissural papule cleaved in two faces. This sign, previously described as* fausse perlèche *(or false angular cheilitis), is also associated with secondary syphilis [19, 21, 22]. The patient indicated that he had never had such lesions in the past. There was no cervical lymphadenopathy or any tegumentary abnormalities. Physical examination was otherwise unremarkable.

The CD4 cell count was 995 cells/mm^3^ and the plasma HIV viral load was consistently below detection limits. A novel VDRL assay was performed and yielded a titer of 1/128. Fluorescent treponemal antibody absorption tests were reactive for IgG and IgM. Laboratory evaluations were otherwise unremarkable. On further history taking, the patient indicated having had unprotected oral and anogenital sexual contact approximately 3 months before the onset of symptoms. A complete clinical remission and resolution of oral lesions (Figure 1(b)) was rapidly recorded after the first of a total of three consecutive weekly administrations of 2.4 million units of intramuscular benzathine penicillin G. A clinical diagnosis of secondary syphilis was then made.

## 3. Discussion

The oral cavity may be involved in primary, secondary, and tertiary stages of syphilis [19, 23, 24]. The mouth is the most common extragenital site of primary syphilis. A chancre will present at the site of inoculation, which can be the lips, tongue, buccal mucosa, tonsils, and oropharynx. Lesions of primary syphilis commonly present as ulcerations that are painless [24]. But painful lesions do occur [23]. These lesions are highly infectious. Primary syphilis of the oral cavity, however, may pass unnoticed by both patient and physician and the untreated lesion will heal regardless of treatment [25, 26].

Tertiary syphilis of the oral cavity may present itself as a gumma or as atrophic luetic glossitis [2]. Gumma is a destructive, granulomatous, usually painless lesion that occurs anywhere in the oral cavity and may enlarge to invade adjacent tissues. In atrophic luetic glossitis, the dorsal aspect of the tongue assumes a smooth and shiny aspect due to atrophy of filiform and fungiform papillae, often with areas of leukoplakia presenting as a homogenous white patch (syphilitic leukoplakia) [1, 27, 28].

Oral lesions of secondary syphilis may be multiple, extremely variable, and nonspecific, both in HIV-negative and HIV-positive patients [18]. They may be accompanied by a concomitant cutaneous eruption [4] and cervical lymphadenopathy [9, 19, 29], which can occasionally dominate the clinical picture [30, 31]. Published case reports and case series testify the outstanding variability of clinical presentation. In a case series of 20 HIV-infected patients with oral secondary syphilis, Ramírez-Amador et al. [18] reported that a mucous patch was the most common oral manifestation (17, 85.5%), followed by shallow ulcers (2, 10%) and macular lesions (1, 5%). In 16 (80%) cases, oral lesions were either the first or most florid clinical sign, whereas in the remaining four patients (20%) they were part of a clinical picture already diagnosed as secondary syphilis. Differently, Hamlyn et al. [9] reported a case series of three patients in whom secondary syphilis presented solely as a tonsillitis. A comprehensive review of the literature shows that oral lesions have been described as solitary or multiple ulcerations [7, 14, 18, 23, 32, 33], as erosions [10, 14], as a bullous-erosive lesion resembling pemphigus vulgaris [34], as macular, papular, and nodular lesions [3, 14, 18], as condylomata lata [6], as leukoplakia-like [35, 36], as oral hairy leukoplakia-like [8, 18] lesions, and as painless nodules on the tongue [37].

Mucous patches are considered the fundamental lesions of oral secondary syphilis. They are frequently described as painful oval or crescentic, slightly raised or shallow erosions. Mucous patches may also present as whitish plaques that may coalesce and form serpiginous lesions, referred to as snail-track ulcers [36]. They present most often on the soft palate, pillars, tongue, and vestibular mucosa [4, 13, 18, 23, 35]. When the dorsal aspect of the tongue is affected, they will efface lingual papillae [11, 21]. Occasionally, mucous patches occur in the ventral tongue [37]. At the angles of the mouth, the mucous patch may present as split papules, as recorded in our patient [19, 21, 22]. Reasonable precautions, such as glove wearing, should be taken when handling such lesions since they are reported to be the most infectious of all [5].

Our patient's painful oral lesions were highly suggestive of secondary syphilis: depapillary erythematous patches on the dorsal aspect of the tongue (also known as* plaques en prairie fauchée*) and a split papule on the oral commissure (also known as* fausse perlèche *or false angular cheilitis) [11, 15, 18–22]. In the present case, these lesions were present on a background of a whitish, nonwipeable hyperkeratotic thickening of the posterior aspect of the tongue. The diagnosis of secondary syphilis was made based on full-history taking, clinical examination, absence of response to azole agents, positive serologic tests for syphilis, and fast remission after initiation of penicillin therapy. Interestingly, signs and symptoms of our patient's illness could only be found in the oral cavity. Histopathology can provide additional evidence of the diagnosis of syphilis [3, 20, 33, 38] and a biopsy would be required had the lesions not subsided completely. Therefore, a decision was made not to perform a biopsy for histopathological examination.

Our patient complained of a burning mouth and dysgeusia that progressed to glossodynia. A burning mouth is occasionally reported as the first presentation of syphilis [39, 40]. Pain is also commonly associated with oral lesions of secondary syphilis. In two recently published cases series (15 and 7 cases), pain was reported by all patients [14, 20] and symptoms were present from 5 to 120 days [20]. However, painless oral lesions may also occur in secondary syphilis [11, 15, 23]. We are unaware of previous reports of dysgeusia and glossodynia as manifestations of oral syphilitic lesions. Dysgeusia is the distortion or perversion of taste [41]. It is caused by diverse conditions such as glossitis, geographic tongue, xerostomia, glossopharyngeal nerve damage, and the use of certain drugs [41]. Glossodynia is the medical term for a painful tongue [41]. The differential diagnosis is also broad. It ranges from obvious causes such as a neoplastic disease, ulcerative conditions, and tongue injury by a dental device, to many other diverse conditions, such as atrophic glossitis of nutritional deficiency and infectious disorders like trichinosis [41]. In the present case, complete clinical remission was rapidly achieved after initiation of penicillin therapy. It seems that our patient's complains of dysgeusia and glossodynia were related to the subacute oral lesions and, consequently, to secondary syphilis.

Due to their transitory nature and heterogeneity of presentation, oral lesions of secondary syphilis are probably underdiagnosed when unaccompanied by tegumentary abnormalities. Udd and Lund [12] recently described a patient who sought relief of sore throat by visiting diverse clinics for more than 6 months. His symptoms were repeatedly attributed to fungal infection or aphthous stomatitis or simply were regarded as stress-related. On examination, erythematous lesions of the soft palate and ulceration of the left buccal mucosa were seen. Rapid improvement was achieved only when syphilis was suspected, serologic tests were requested and appropriate treatment was instituted [12]. A similar case in which a bleeding ulcer in the lower lip remained undiagnosed for 5 months was described by Strieder et al. [33]. These cases highlight that a high level of suspicion of syphilis should be exercised when dealing with patients with oral lesions.

In summary, the present case report emphasizes the importance of considering syphilis in the differential diagnosis of unexplained oral lesions. Failure to recognize syphilis could have devastating consequences. If not suspected and left untreated, such oral lesions will undergo spontaneous remission and enter a latent stage. Potentially life-threatening complications of tertiary syphilis could then ensue. The long periods of latency of syphilis could give the false impression that symptomatic treatments, such as topical corticosteroids, were successful. Therefore, it is essential that health care providers be familiar with the full spectrum of clinical presentations of syphilis.
